# Maximum power point tracking of PEMFC based on hybrid artificial bee colony algorithm with fuzzy control

**DOI:** 10.1038/s41598-022-08327-5

**Published:** 2022-03-12

**Authors:** Liping Fan, Xianyang Ma

**Affiliations:** 1 College of Information Engineering, Shenyang University of Chemical Technology, Shenyang, 110142 China; 2 Key Laboratory of Collaborative Control and Optimization Technology of Industrial Environment and Resource of Liaoning Province, Shenyang University of Chemical Technology, Shenyang, 110142 China

**Keywords:** Energy science and technology, Engineering, Mathematics and computing

## Abstract

Maximum power point tracking (MPPT) is an effective method to improve the power generation efficiency and power supply quality of a proton exchange membrane fuel cell (PEMFC). Due to the inherent nonlinear characteristics of PEMFC, conventional MPPT methods are often difficult to achieve a satisfactory control effect. Considering this, artificial bee colony algorithm combining fuzzy control (ABC-fuzzy) was proposed to construct a MPPT control scheme for PEMFC. The global optimization ability of ABC algorithm was used to approach the maximum power point of PEMFC and solve the problem of falling into local optimization, and fuzzy control was used to eliminate the problems of large overshoot and slow convergence speed of ABC algorithm. The testing results show that compared with perturb & observe algorithm, conductance increment and ABC methods, ABC-fuzzy method can make PEMFC obtain greater output power, faster regulation speed, smaller steady-state error, less oscillation and stronger anti-interference ability. The MPPT scheme based on ABC-fuzzy can effectively realize the maximum power output of PEMFC, and plays an important role in improving the service life and power supply efficiency of PEMFC.

## Introduction

About 85% of the energy needed for human survival and development comes from fossil fuels, which are 57% of the global carbon emission sources^1^. To achieve sustainable development, more and more attention has been paid to the development and utilization of renewable and clean energy^2^.

As a kind of power generation equipment that directly converts chemical energy into electric energy, fuel cell is considered to be one of the most potential green energies. Among them, proton exchange membrane fuel cell (PEMFC) is the best alternative to traditional power supply due to its advantages of simple structure, fast start, high power density, low noise, zero pollution and so on^3–5^. PEMFC has great application potential in electric vehicles, distributed power generation, portable power system, islanded microgrid, aerospace equipment, etc., and plays an important role in alleviating environmental pressure and rebuilding energy consumption structure because of its outstanding environment-friendly characteristics^6,7^. However, its disadvantages such as short service life, fast performance decay and high maintenance cost have not been well solved, which hinder their further wide popularization and applications^8,9^.

Advanced control technology is an alternative solution to improve the overall performance of an energy system. Among them, the Maximum power point tracking (MPPT) algorithm is recognized as an effective method to solve the problem of power output efficiency of power generation system, which has been widely used in photovoltaic power generation system (PV)^10–13^, wind power generation system^14,15^, and wind solar hybrid power system^16^. In recent years, the application of MPPT in fuel cell system has been reported^17–19^.

The output characteristics of PEMFC are nonlinear, which are affected by temperature, oxygen partial pressure, hydrogen partial pressure, membrane water content, stoichiometry, etc. However, under each specific condition, the PEMFC has only one unique working point corresponding to the maximum power point (MPP)^20^. Therefore, tracking and stable operation at the maximum power point is very important to improve the energy efficiency of PEMFC.

The commonly used MPPT methods mainly include constant voltage (CVT), perturb & observe algorithm (P&O), conductance increment (INC), hill climbing (HC), etc.^21^. P&O is usually used to track the maximum power of PEMFC, but the tracking speed is slow and misoperation is easy to occur in the case of sudden changes^22^. Ahmadi et al. proposed an MPPT for PEMFC based on the particle swarm optimization (PSO) and PID controller (PSO-PID), but there are problems of local optimization and too slow optimization speed^23^. Harrag et al. addressed a variable step fuzzy based MPPT controller, but the setting of fuzzy rules and proportional quantization factors needed the support of practical experience^24^. Harrag et al. proposed a single sensor variable step size MPPT method for PEMFC, although the steady state performance could meet the demand, the dynamic performance was not particularly satisfactory^25^. Liu et al. designed a fractional order high pass filter (FOHPF) to improve traditional extremum seeking control (ESC), but the response speed was difficult to guarantee^26^. In the past decade, there has been a great development of maximum power point tracking controllers for fuel cell power systems. Mallick et al. proposed an adaptive hybrid controller based on artificial neural network (ANN), which successfully tracked MPP with less oscillation under variable temperature^27^. Fathy et al. proposed a strategy based on salp swarm algorithm (SSA), which has high reliability and high efficiency when extracting the maximum power of PEMFC^28^. Souissi used an adaptive sliding mode controller for MPPT of PEMFC and chattering reduction of 85% has been achieved^29^.

Compared to the difficulty of improving the efficiency of a converter or the conversion rate of a PEMFC, using some advanced control strategy to improve the efficiency of MPPT control is a relatively simple, fast and more efficient way^30,31^. However, the commonly used MPPT methods still have some problems, such as slow tracking speed, large steady-state fluctuation, poor anti-interference ability, etc., which need to be further improved to raise the tracking accuracy and speed.

In recent years, evolutionary algorithms (EAs) have been used for optimization problems. EA is a global search optimization technique based on biological evolution mechanisms such as natural selection and genetic variation, mainly including genetic algorithm (GA), particle swarm optimization (PSO), ant colony optimization (ACO), gray wolf optimization (GWO), cuckoo search (CS), artificial bee colony (ABC)^32–34^, etc. Since the EAs are stochastic search algorithms, their probability of finding approximate best solutions from finite set of solution space at the early stage of the optimization process is very high. Ma et al. proposed a new large-scale multi-objective EA to solve the large-scale multiobjective and many-objective optimization problems^35^. An orthogonal learning framework for brain storm optimization (OLBSO) was proved very powerful in optimizing complex functions^36^. Younas et al. introduced EA to solve the sensor selection problem in Internet of Thing (IoT) systems^37^. There have been many studies on EAs for gene selection and detection of fatal diseases^38–41^. EAs were also used for reservoir operation^42^, dynamic optimization of biodiesel production process^43^, vehicle route planning^44^, traffic signal control^45^, assembly line balancing^46^, MPPT of hybrid renewable energy system^47^, etc. EAs will play an increasingly important role in solving practical optimization problems in a wide range of science and engineering^48^, and it provides a broader idea for MPPT control of PEMFC.

Among EAs, ABC is an optimization algorithm based on the intelligent behavior of honey bee swarm^49^. Compared with other algorithms based on swarm intelligence and population such as GA, PSO and Particle Swarm Inspired Evolutionary Algorithm (PS-EA), ABC has the advantages of relative simplicity, strong robustness, fast convergence, fewer setting parameters and high flexibility, and is considered to be an excellent global optimization algorithm^50–54^. In view of these advantages, ABC has been applied to numerous benchmark problems and real-world problems^55^. Zirkohi employed ABC algorithm to optimize the parameters of the two fractional-order PI controllers of induction motor and achieved better control effect^56^. Zhu et al. used ABC algorithm in solving examination and course timetabling problems, which significantly reduced computational costs^57^. Chen et al. introduced ABC algorithm in optimization of transfer station selection and train timetables for road–rail intermodal transport network^58^.

In consideration of the advantages of simplicity and robustness of ABC algorithm, it was selected to design a new MPPT scheme for PEMFC. In order to overcome the defects such as large overshoot during startup in ABC, this paper proposed a hybrid ABC algorithm integrated with fuzzy control (ABC-fuzzy) for MPPT control of PEMFC to solve the problem of accurate maximum power tracking, and its control effect was compared with the traditional P&O algorithm, variable step INC and common ABC. ABC-fuzzy algorithm has faster tracking speed, smaller steady-state error and stronger anti-interference ability, which effectively improves the MPPT control effect of PEMFC.

## Mathematical model of PEMFC

PEMFC is a kind of electrochemical device, which can directly convert the chemical energy of fuel (hydrogen) and oxidant (oxygen) into electrical energy. The main components of PEMFC are anode, cathode and proton exchange membrane. The hydrogen is decomposed into protons and electrons in the anode. The protons move to the cathode through the proton exchange membrane and react with oxygen in the cathode to form water, while the electrons are transferred to the cathode through an external circuit and thus the terminal voltage is produced.

The stack total voltage can be represented by a controlled voltage source in series with a constant resistance^59^. The controlled voltage source (*E*) is described by:

1 $$ E=E_{{{\text{oc}}}} - NA\ln \left( {\frac{{i_{{{\text{FC}}}} }}{{i_{{\text{o}}} }}} \right) \cdot \frac{1}{{{{{\text{s}}T_{{\text{d}}} } \mathord{\left/ {\vphantom {{{\text{s}}T_{{\text{d}}} } 3}} \right. \kern-\nulldelimiterspace} 3} + 1}} $$

in which *E*_oc_ is the open circuit voltage (V); *N* is the number of cells; *A* is the Tafel slope (V); *i*_o_ is the exchange current (A); *T*_d_ is the response time (at 95% of the final value) (sec); and *i*_FC_ denotes the output current (A).

The output voltage of the PEMFC stack can be described as:

2 $$ U_{{{\text{FC}}}} = E - R_{{{\text{ohm}}}} i_{{{\text{FC}}}} $$

where *R*_ohm_ is the internal resistance (Ω); *U*_FC_ denotes the output voltage (V).

*E*_oc_, *i*_o_ and *A* are described as follows:

3 $$ E_{{{\text{oc}}}} = K_{{\text{c}}} E_{{\text{n}}} $$

4 $$ i_{{\text{o}}} = \frac{{zFk(P_{{{\text{H}}_{2} }} + P_{{{\text{O}}_{2} }} )}}{Rh}\exp \left( {\frac{ - \Delta G}{{RT}}} \right) $$

5 $$ A = \frac{RT}{{z\alpha F}} $$

where *E*_n_ is the Nernst voltage (V); *K*_c_ is the voltage constant at nominal condition of operation; *R* is a gas constant (J/(mol K)); *F* is the Faridy's constant (s/mol); *z* is the number of moving electrons; *k* is Boltzmann’s constant (J/K); *h* is Planck’s constant (Js); *P*_H2_ is the partial pressure of hydrogen inside the stack (bar); *P*_O2_ is the partial pressure of oxygen inside the stack (bar); ∆*G* is the activation energy barrier (J); *T* is the operating temperature (K), and *α* is the charge transfer coefficient.

The partial pressures and the Nernst voltage can be calculated by:

6 $$ P_{{{\text{H}}_{2} }} = (1 - \lambda_{{{\text{H}}_{{2}} }} )xP_{{{\text{Fuel}}}} $$

7 $$ P_{{{\text{O}}_{2} }} = (1 - \lambda_{{{\text{O}}_{{2}} }} )yP_{{{\text{Air}}}} $$

8 $$ E_{{\text{n}}} = 1.229 + (T - 298)\frac{ - 44.43}{{zF}} + \frac{RT}{{zF}}\ln (P_{{{\text{H}}_{2} }} P_{{{\text{O}}_{2} }}^{{\tfrac{1}{2}}} ) $$

where *x* denotes the percentage of hydrogen in the fuel (%); *y* denotes the percentage of oxygen in the oxidant (%); *P*_Fuel_ denotes the absolute supply pressure of fuel (bar); *P*_Air_ denotes the absolute supply pressure of air (bar); *λ*_H2_ and *λ*_O2_ are the conversion rates of hydrogen and oxygen that can be represented as:

9 $$ \lambda_{{{\text{H}}_{{2}} }} { = }\frac{{60000RTi_{{{\text{FC}}}} }}{{zFP_{{{\text{Fuel}}}} Q_{{{\text{Fuel}}}} x}} $$

10 $$ \lambda_{{{\text{O}}_{2} }} { = }\frac{{60000RTi_{{{\text{FC}}}} }}{{2zFP_{{{\text{Air}}}} Q_{{{\text{Air}}}} y}} $$

where *Q*_Fuel_ denotes the fuel flow rate (L/min); *Q*_Air_ denotes the air flow rate (L/min).

The output power used to measure the power supply capacity of a PEMFC stack can be calculated as follows:

11 $$ P = i_{{{\text{FC}}}} U_{{{\text{FC}}}} $$

**Figure 1:**
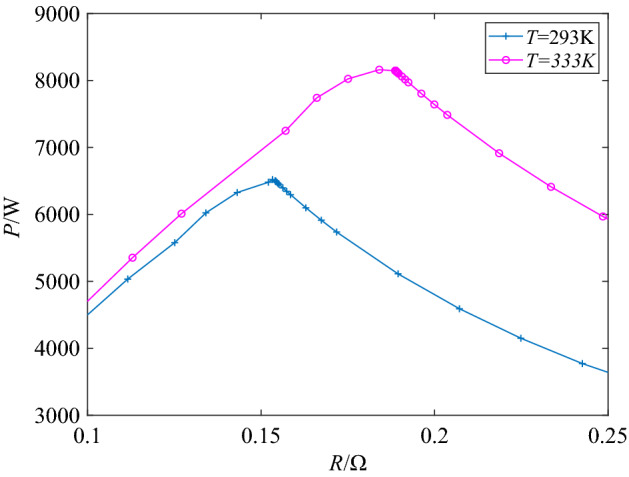
*P-R* characteristics of the used PEMFC.

A simulation module of 6 kW fuel cell stack built according to the above model is used to simulate the PEMFC stack in this study. The main parameters are shown in Table 1. The output power curves of PEMFC with the change of load resistance under two different temperature conditions are shown in Fig. 1.

**Table 1:** Main parameter used the model.

Symbol	Physical meaning	Value	Unit
*F*	Faridy's constant	96,485	As/mol
*P*_Air_	Air pressure	1	bar
*R*	gas constant	8.3145	J/(molK)
*A*	The battery area	0.0240	m^2^
*z*	moving electrons	2	–
*k*	Boltzmann’s constant	1.38 × 10^−23^	J/K
*h*	Planck’s constant	6.626 × 10^−34^	Js
*y*	Oxygen concentrations	21	%
*x*	Hydrogen concentration	99.95	%
*N*	Number of Batteries	65	–
*P*_Fuel_	Fuel pressure	1.5	bar

It can be clearly seen from the curves in Fig. 1 that there is indeed an obvious peak on each operation curve, which indicates that the maximum power point is inevitable. According to the relevant circuit knowledge, the output power of a power supply reaches its maximum value when the the load resistance is equal to its internal resistance. It can be seen that when operating at 333 K, the maximum output power (*P*_max_) value of the PEMFC is about 8162 W, and the load resistance at the maximum power is about 0.18 Ω, which means that the internal resistance of the PEMFC at 333 K is about 0.18 Ω. When the temperature changes to 293 K under the same other conditions, the maximum output power is about 6520 W and the corresponding internal resistance is about 0.15 Ω. As long as the external load resistance can always be equal to the internal resistance through effective methods, this maximum power point will be tracked in real time to keep the PEMFC at maximum power output all the times.

In addition, it can also be seen from the *P-R* curves that temperature has an impact on the output power of PEMFC. The maximum output power and internal resistance of a PEMFC are different at different temperatures.

## Scheme design of MPPT

### Structure of control system

**Figure 2:**
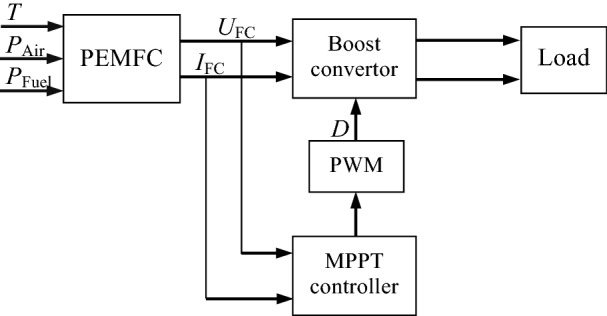
Diagram of MPPT control system of PEMFC.

Figure 2 displays the diagrammatic sketch of the MPPT control system of PEMFC with Boost converter, which is mainly composed of PEMFC, boost converter, MPPT controller, PWM generator and external load resistance. Because the Boost converter has the advantages of simple structure, easy control and voltage amplification, it is used in the system to improve the output voltage and as a regulator to realize the MPPT control scheme. The output of PWM generator is a series of square waves with different duty cycle, which is used to control the ON and OFF of the Boost convertor. The output duty cycle of the PWM controller is represented by *D*.

For the system shown in Fig. 2, the equivalent load resistance of PEMFC can be expressed according to the knowledge of power electronic technology as:

12 $$ R_{{{\text{eq}}}} = \frac{U}{I} = (1 - D)^{2} R_{{\text{L}}} $$

in which *R*_eq_ is the equivalent load resistance of PEMFC, and *R*_L_ denotes the actual load resistance, that is, the load resistance at the output of the Boost converter.

It is well known that the output power of an electric source can reach its maximum if and only if its external resistance is equal to its internal resistance. According to Eq. (12), the purpose of adjusting the equivalent load resistance can be achieved by adjusting the duty cycle of the Boost converter. Therefore, by adjusting the duty cycle, the equivalent load resistance of PEMFC can be equal to its internal resistance, so as to achieve the purpose of maximum power output.

### P&O algorithm

**Figure 3:**
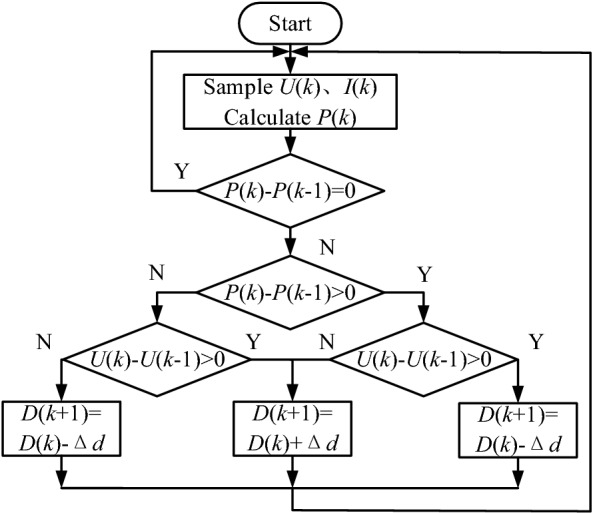
Flow chart of P&O algorithm of MFC.

P&O is the most widely used MPPT method at present. Its basic principle is to apply a positive disturbance voltage to the fuel cell, sample its output voltage and current, and calculate the change rate of its voltage and power. If the output power increases after the disturbance, it indicates that the direction of the disturbance voltage is the direction of the increase of the power output, and the disturbance should be continued in this direction; otherwise, the disturbance should be in the opposite direction. According to the changing direction of voltage and power, the disturbance voltage is continuously applied to the fuel cell until the maximum output power is approached gradually, so as to realize the MPPT. The flowchart of P&O algorithm is shown in Fig. 3, in which “*k*” denotes the sampling point, $\Delta d$ stands for the increment of duty cycle.

P&O has the advantages of simple calculation and convenient implementation, but it can only keep the system oscillating infinitely near the maximum power point, and can not accurately track the maximum power point, so it can not effectively solve the problems of tracking accuracy and speed.

### Variable step INC MPPT

The incremental conductance (INC) method determines whether the maximum power point is tracked according to the slope of the voltage–power curve of PEMFC. If d*P*/d*U* = 0, the operating point is at the maximum power point and the operating voltage needs to be kept unchanged; if d*P*/d*U* < 0, the working point is on the right side of the maximum power point, and the working voltage needs to be reduced; if d*P*/d*U* > 0, the working point is on the left side of the maximum power point, and the working voltage needs to be increased.

When the INC method is used for MPPT, the tracking speed of the system is determined by the step size. When the step size is large, the tracking speed is fast, but it is difficult to keep it after reaching the maximum power point, and it is easy to produce concussion. On the contrary, when the step size is small, the tracking is stable after reaching the maximum power point, but the tracking speed is relatively slow. The step size selection needs to balance tracking speed and tracking accuracy, so it is very difficult to select appropriate step size.

By observing the voltage–power curve, a variable step size method is proposed. Considering the MPPT control system shown in Fig. 2, the duty cycle of Boost converter is adjusted by the following formula:

13 $$ D(k) = D(k - 1) \pm \upsilon \left| {\frac{{{\text{d}}P}}{{{\text{d}}U}}} \right| $$

where *D*(*k*) represents the duty cycle of the boost converter at the sampling time *k*; *υ* is the speed factor used for adjusting the step size. To ensure the convergence of the MPPT update rule, the speed factor must satisfy the following relationship:

14 $$ \upsilon < \Delta d_{\max } \left| {\frac{{{\text{d}}P}}{{{\text{d}}U}}} \right| $$

where Δ*d*_max_ indicates the maximum change of duty cycle in the previous sampling period.

When Eq. (14) is satisfied, the system can work in variable step mode, and the greater the *υ*, the faster the response^60,61^. If Eq. (14) cannot be satisfied, the system will work with the constant step of Δ*d*_max_. The variable step size INC (VS-INC) can effectively improve the tracking speed and accuracy, but the selection of speed factor and Δ*d*_max_ is specific, so different speed factors and Δ*d*_max_ need to be set in different cases, otherwise the tracking effect will fail to meet the requirements, resulting in the tracking dead zone.

### ABC-based MPPT

Artificial Bee Colony (ABC) algorithm is an optimization algorithm inspired by honeybee's nectar gathering behavior and designed based on random population. Because of its simplicity, efficiency, robustness and global search capability, it is proved to be one of the most effective naturally-inspired algorithms for dealing with unconstrained and constrained single-objective or multi-objective global optimization problems.

In the ABC algorithm, all the bees are divided into three groups: employed bees, onlookers and scouts. The three kinds of honeybees exploit nectar sources through division of labor and cooperation, and constantly update the location of nectar sources through marking and sharing to find the optimal nectar sources. The location of the nectar source corresponds to the feasible solution of the optimization problem, and the quality of the nectar source is measured by the fitness value of the optimization problem. The main process of ABC algorithm includes^62–65^:

(1) *Initialization stage* Set the number of various bee colonies, optimization range, dimension and maximum iterations.

When ABC algorithm is used to optimize the duty cycle *D* in this study, the initial number of the employed bees and onlooker bees are set to be 50 and 30 respectively, the dimension is set to 1, the maximum number of iterations is set to 10, and the optimization range is set as (0.85, 1).

(2) *Employed bee phase* The employed bees searched for a new food source near their current food source.

To produce a candidate food position from the old one, the new solution is compared with the current solution, and the fitness is calculated according to the following formula:

15 $$ V_{ij} = X_{ij} + \varphi_{ij} (X_{ij} - X_{kj} ) $$

where *V*_ij_ denotes the new location of food source; *X*_ij_ denotes the current food source; *i* $\in${1, 2,…, *S*_*N*_} is the position of the food source; *k* $\in${1, 2,…, *S*_*N*_} and *j* $\in${1, 2,…, *M*} are randomly chosen indexes, *S*_*N*_ is the number of solutions in the colony (that is, the size of the swarm), *M* is the dimension of the problem; and *φ*_*ij*_ is a random number in the range [− 1,1].

(3) *Onlooker bee phase* The onlookers choose the food source after sharing information of employed bees and determine the amount of nectar. Some better solutions are selected according to a probability which is computed by:

16 $$ p_{i} = \frac{{f_{i} }}{{\sum\limits_{i = 1}^{{S_{N} }} {f_{i} } }} $$

in which *f*_*i*_ is the fitness function value of the *i*^th^ solution.

(4) *Scout bee phase* In each iteration, the scout bees monitor the changes of each solution in the swarm. If a food source cannot be updated through a predetermined cycle, it will be removed from the population and the employed bees of the food source will become scout, and they use the following equation to find a new random food source location:

17 $$ X_{ij} = X_{\min j} + {\text{rand}}[0,1](X_{\max j} - X_{\min j} ) $$

### ABC-Fuzzy MPPT

**Figure 4:**
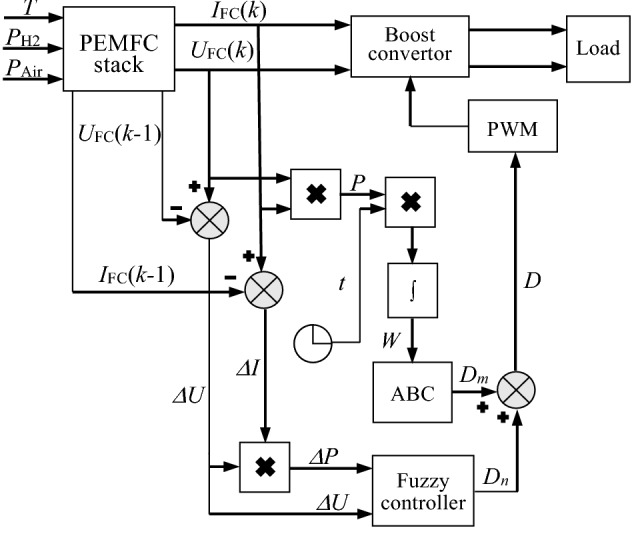
Structure diagram of ABC-fuzzy MPPT.

Although ABC shows good search performance, it still has the disadvantages of slow convergence speed and low solving accuracy^66^. To solve these problems, a MPPT scheme based on ABC algorithm integrated with fuzzy control (ABC-fuzzy) is proposed. The structure diagram of the ABC-fuzzy MPPT control system is shown in Fig. 4.

The integral of power multiplied by time is introduced as the intermediate variable as shown in the following formula:

18 $$ W = \int_{{t_{0} }}^{{t_{1} }} {t\left| P \right|} {\text{d}}t $$

where *W* is the electric energy produced by the fuel cell. In the same time period, the greater the power generation energy *W*, the greater the output power *P*. Therefore, it is used as the fitness to compare and judge the choice of duty cycle.

The power error Δ*P* and the voltage error Δ*U* are respectively used as the inputs of the fuzzy controller, and its output is used as one of the components (*D*_n_) of the duty cycle. The basic domain of Δ*P* and Δ*U* are designed as [− 100, 100] and [− 10, 10], respectively; and the basic domain of *D*_n_ is designed as [− 8, 8]. In order to achieve good control effect, the fuzzy domains of Δ*P* and Δ*U* are divided into five fuzzy subsets, namely {NB, NS, ZE, PS, PB}, and the fuzzy domains of *D*_n_ are divided into seven fuzzy subsets, namely {NB, NM, NS, ZE, PS, PM, PB}.

The detailed steps of ABC-fuzzy algorithm are shown in Table 2, and the fuzzy control rules are designed as shown in Table 3.

**Table 2:** Steps for ABC-fuzzy algorithm.

	Operation
Step 1	**Initialization:** Employed bees = 30 Onlooker bees = 20 Dimension = 1 Maximum iterations = 10 Optimization range = (0.85,1) Initialize population parameters *D*_1_…*D*_*r*_ Take a random number in *D*_1_…*D*_*r*_ as the output *D*_m_
Step 2	Call *D*_m_ and *D*_n_, the output of the fuzzy controller for superposition, and its output is used to adjust the duty cycle to *D*. Measure *U*_FC_ and *I*_FC_, calculate the energy *W,* and use it as the fitness value corresponding to *D*_m_ in ABC
Step 3	**Employed Bee Phase:** Update *D*_m_ according to Eq. (15) and repeat step 2
Step 4	Compare the fitness. If the fitness is better, update the optimal *D*_m_ and the optimal fitness, otherwise update the stagnation times
Step 5	The probability of selecting *D*_m_ is calculated by roulette
Step 6	**Onlooker bee phase:** Select *D*_m_ according to probability, and generates a new *D*_m_ according to Eq. (15)
Step 7	Repeat steps 2 and 4 to update *D*_m_ continuously according to the greedy strategy
Step 8	**Scout bee phase:** Compare the stagnation times. If it exceeds the limit, select a new *D*_m_ according to Eq. (17) to replace the overrun *D*_m_
Step 9	Repeat step 2 and record the updated *D*_m_ and fitness
Step 10	Evaluate *D*_m_. If *D*_m_ meets the optimal condition, then end the cycle, otherwise return to step 3

**Table 3:** The designed fuzzy rules.

		*ΔP*				
		NB	NS	ZE	PS	PB
*ΔU*	NB	PB	PM	NS	NM	NB
	NS	PM	PS	NS	NS	PM
	ZE	NM	NS	ZE	PS	PM
	PS	NM	NS	PS	PS	PM
	PB	NB	NM	PS	PM	PB

## System simulation

A simulation model of MPPT control system of PEMFC with the structure shown in Fig. 2 was established in MATLAB simulation environment. In this simulation, the rated power of the PEMFC was 6 kW, and the load resistance was *R*_L_ = 70 Ω. The main parameters of the Boost converter were as follows: the inductor was *L* = 3 mH and the capacitor was *C* = 0.47 mF, and the sampling frequency was 20 kHz.

Four MPPT control algorithms, including P&O, variable step INC (VS-INC), ABC and ABC-Fuzzy, were applied to this PEMFC system respectively. In order to facilitate the comprehensive performance evaluation of the controlled PEMFC system, these different control schemes were tested under the conditions of temperature change and inlet pressure change respectively.

### Operation under inlet pressure variation

**Figure 5:**
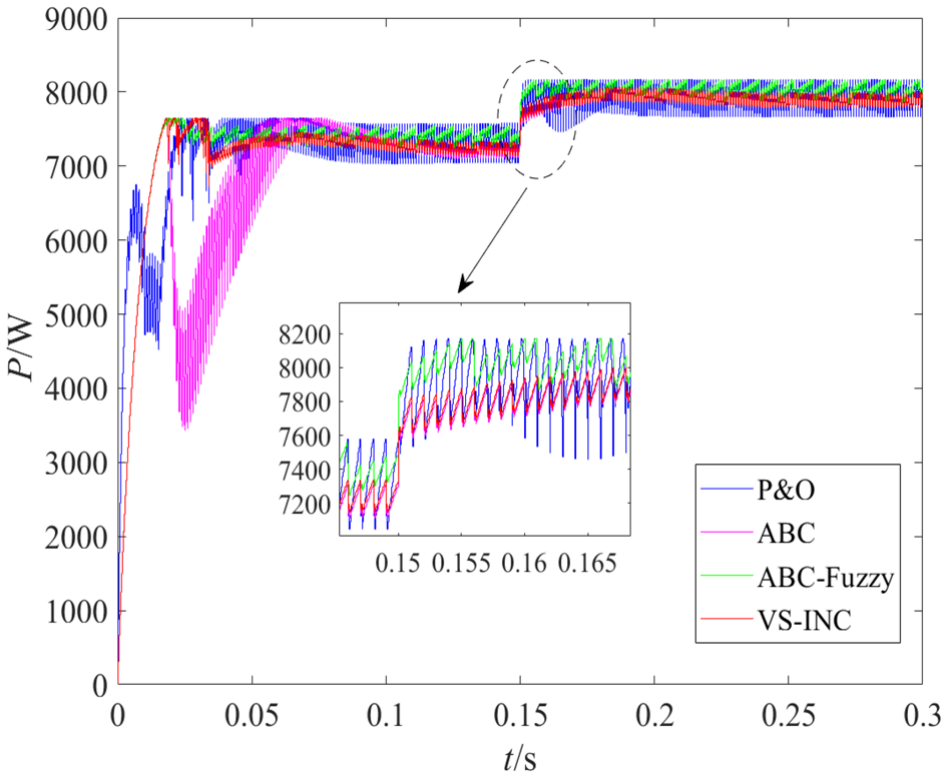
Power curves under pressure change.

In this test, the operating temperature of the PEMFC system was kept at 333 K but the inlet fuel pressure and air pressure jumped from the initial value of 0.5 bar to 1.5 bar and 0.5 bar to 1 bar respectively at 0.15 s. Under such conditions, the main performance indexes are listed in Table 4, and the output power variation curves of the PEMFC systems with different control schemes are shown in Fig. 5. In Table 4, *S*_tr_ denotes the tracking speed (s); *P*_S_ denotes the steady output power (W); *O*_max_ denotes the maximum overshoot (W); *A*_SA_ denotes the steady amplitude (W); and Fl denotes fluctuation (%).

**Table 4:** Experimental data under pressure change.

Algorithm	*S*_tr_	*P*_S_	*O*_max_	*A*_SA_	Fl
P&O	0.09	7210	2785	269	37.3
VS-INC	0.10	7242	919	101	1.39
ABC	0.13	7311	3848	102	1.40
ABC-fuzzy	0.03	7524	257	93	1.24

It can be seen that, among the four different MPPT control algorithms, ABC-fuzzy algorithm obtained the fastest tracking speed, maximum output power, weakest amplitude fluctuation and minimum steady-state error. In the start-up stage, the tracking speed of P&O, VS-INC, ABC and ABC-fuzzy algorithm were 0.09 s, 0.10 s, 0.13 s and 0.03 s respectively, that is, the tracking speed of ABC-fuzzy was 67%, 70% and 77% faster than that of P&O, VS-INC and ABC respectively. The ABC algorithm had the slowest tracking speed and biggest overshoot in the start-up stage, which further confirmed its defect of "slow convergence speed" mentioned above, and also proved that it is not feasible to use ABC algorithm alone for MPPT control. The ABC-fuzzy algorithm not only improved the tracking accuracy, but also greatly reduced the tracking adjustment time. In addition, when the intake pressure changed, ABC-fuzzy algorithm could adjust the tracking state in time, re-track and stabilize at the new maximum power point with the fastest speed, the smallest error and the weakest fluctuation.

### Operation under varying temperature

**Figure 6:**
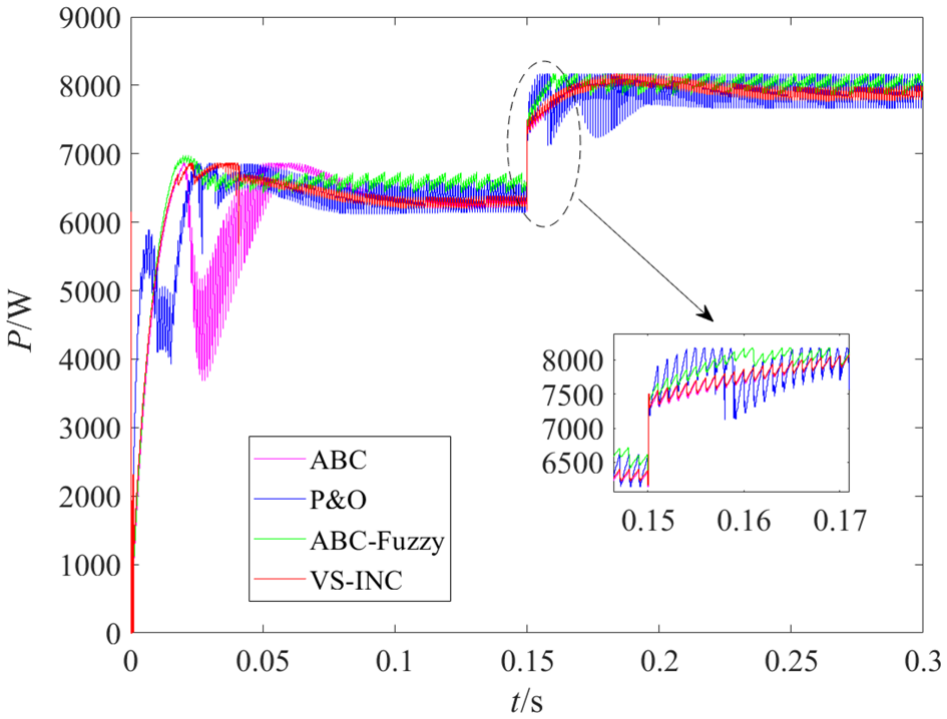
Power curves with variable temperature.

During this test run, the intake pressures of PEMFC remained unchanged at the values shown in Table 1. The initial temperature was set at 293 K, and then it changed to 333 K at 0.15 s. The output power curves in this case are shown in Fig. 6.

By comparing the operation results under the action of four MPPT methods, it can be found that ABC-fuzzy algorithm has obvious advantages in tracking accuracy and tracking speed. Before encountering temperature variation, ABC-fuzzy algorithm could track the MPP after about 0.03 s of adjustment and stabilized at the MPP with weak fluctuation, while the time required for the conventional ABC algorithm to approach MPP was about 0.12 s, which was four times of that required by ABC-fuzzy. It is thus clear that ABC-fuzzy algorithm effectively overcame the problem of slow convergence speed of ABC.

On the other hand, under operating conditions at 293 K, the actual steady output power tracked by ABC-fuzzy was about 6510 W, and the stable fluctuation percentage was less than 1.2%, while the output power tracked by9 ABC algorithm was about 6280 W, which was far lower than the actual MPP. The output power generated by PEMFC controlled by P&O algorithm was about 6300 W, but the steady-state fluctuation was greater than 3% and the stabilization time was about 0.09 s, which was three times that of ABC-fuzzy algorithm. The output power obtained by VS-INC algorithm fluctuated slightly, but the output power was about 6300 W, which was far lower than the actual maximum power, and the adjustment time was about 0.12 s, which was about 4 times that of ABC-fuzzy algorithm. When temperature changed, ABC-fuzzy algorithm also presented best results in tracking speed and tracking accuracy. So, the hybrid ABC algorithm with fuzzy control is effective to realize the MPPT control of PEMFC, which is a feasible scheme to improve the power supply quality and prolong the service life of PEMFC.

The proposed ABC-fuzzy algorithm was compared with some other new MPPT methods such as Fireworks algorithm (FWA), Particle swarm optimization (PSO), Bat algorithm (BA), Grasshopper optimization (GO), Flower pollination algorithm (FPA), Squirrel search algorithm (SSA), Gray wolf optimization (GWO), Seagull optimization algorithm (SOA), Water cycle algorithm (WCA) and Sliding mode control (SMC), and the main indicators were shown in Table 5. The first four rows of the table correspond to the experimental results of the four schemes involved in this paper before the temperature changes, which is consistent with the first stage of Fig. 6. All PV systems in the table work under uniform light conditions.

**Table 5:** Comparison of ABC-fuzzy with some other methods.

Algorithm	Object	Tracking speed (s)	*η* (%)	References
P&O	PEMFC	0.09	96.6	This work
VS-INC	PEMFC	0.12	96.6	This work
ABC	PEMFC	0.12	96.3	This work
ABC-fuzzy	PEMFC	0.03	99.8	This work
PSO	PV	–	92.5	^67^
FWA	PV	8.90	99.1	^68^
BA-ANFIS	PV	–	99.9	^69^
SSA	PV	0.66	99.5	^70^
GO-fuzzy	PV	0.13	99.8	^71^
Fuzzy-SMC	PV	–	97.5	^72^
FPA	PV	–	99.7	^73^
SOA	PV	–	99.4	^74^
GWO-fuzzy	PV	–	91.0	^75^
WCA	PV	0.20	99.8	^76^

In Table 5, *η* is the MPPT Efficiency that can be obtained by the following formula:

19 $$ \eta { = }\frac{{P_{{{\text{ms}}}} }}{{P_{{{\text{max}}}} }} \times 100{\text{\% }} $$

where *P*_ms_ represents the steady-state output power tracked by MPPT algorithm; *P*_max_ represents the actual maximum power of PEMFC. By comparing the data in the table, it can be seen that the overall performance of MPPT algorithm based on ABC-Fuzzy is superior. The tracking efficiency of ABC-Fuzzy algorithm is basically one of the best among all the comparison methods, and it has a significant advantage in the tracking speed. This further proves the feasibility of the MPPT scheme based on ABC-Fuzzy algorithm.

## Conclusion

In order to improve the power generation efficiency and power supply quality of PEMFC, it is an effective method to keep it in the maximum power output state through advanced control. Because of the nonlinear characteristics of PEMFC, the conventional MPPT method is difficult to achieve satisfactory results. The proposed ABC-fuzzy algorithm can not only overcome the problems of large tracking error and poor anti-disturbance ability existing in P&O and other conventional MPPT algorithms, but also overcome the problems of slow tracking speed and large overshoot existing in conventional ABC algorithm. Compared with MPPT methods based on P&O, VS-INC and ABC, ABC-fuzzy algorithm has the advantages of fastest tracking speed, weakest fluctuation, largest output power and minimum steady-state error. Compared with some existing advanced MPPT methods, ABC- fuzzy also has certain advantages in tracking speed and tracking accuracy.

In the future research, we will focus on the implementation of the proposed ABC-fuzzy algorithm in the actual PEMFC system and some other new energy systems, and conduct more in-depth research on MPPT algorithm to further improve the tracking accuracy, tracking speed and application effect of MPPT.
